# Safety of accelerated hypofractionated whole pelvis radiation therapy prior to high dose rate brachytherapy or stereotactic body radiation therapy prostate boost

**DOI:** 10.1186/s13014-021-01976-2

**Published:** 2022-01-20

**Authors:** Christina Phuong, Jason W. Chan, Lisa Ni, Phillip Wall, Osama Mohamad, Anthony C. Wong, I.-Chow Hsu, Albert J. Chang

**Affiliations:** 1 Department of Radiation Oncology, University of California San Francisco, San Francisco, USA; 2 Department of Radiation Oncology, University of California Los Angeles, Los Angeles, USA; 3 UCLA Radiation Oncology, 200 Medical Plaza Suite B265, Los Angeles, CA 90024 USA

**Keywords:** Hypofractionation, Prostate cancer, Nodal radiotherapy, Pelvic radiotherapy, HDR brachytherapy

## Abstract

**Background:**

To evaluate acute and late genitourinary and gastrointestinal toxicities and patient reported urinary and sexual function following accelerated, hypofractionated external beam radiotherapy to the prostate, seminal vesicles and pelvic lymph nodes and high dose rate (HDR) brachytherapy or stereotactic body radiation therapy (SBRT) prostate boost.

**Methods:**

Patients at a single institution with NCCN intermediate- and high-risk localized prostate cancer with logistical barriers to completing five weeks of whole pelvic radiotherapy (WPRT) were retrospectively reviewed for toxicity following accelerated, hypofractionated WPRT (41.25 Gy in 15 fractions of 2.75 Gy). Patients also received prostate boost radiotherapy with either HDR brachytherapy (1 fraction of 15 Gy) or SBRT (19 Gy in 2 fractions of 9.5 Gy). The duration of androgen deprivation therapy was at the discretion of the treating radiation oncologist. Toxicity was evaluated by NCI CTCAE v 5.0.

**Results:**

Between 2015 and 2017, 22 patients with a median age of 71 years completed accelerated, hypofractionated WPRT. Median follow-up from the end of radiotherapy was 32 months (range 2–57). 5%, 73%, and 23% of patients had clinical T1, T2, and T3 disease, respectively. 86% of tumors were Gleason grade 7 and 14% were Gleason grade 9. 68% and 32% of patients had NCCN intermediate- and high-risk disease, respectively. 91% and 9% of patients received HDR brachytherapy and SBRT prostate boost following WPRT, respectively. Crude rates of grade 2 or higher GI and GU toxicities were 23% and 23%, respectively. 3 patients (14%) had late or persistent grade 2 toxicities of urinary frequency and 1 patient (5%) had late or persistent GI toxicity of diarrhea. No patient experienced grade 3 or higher toxicity at any time. No difference in patient-reported urinary or sexual function was noted at 12 months.

**Conclusions:**

Accelerated, hypofractionated whole pelvis radiotherapy was associated with acceptable GU and GI toxicities and should be further validated for those at risk for harboring occult nodal disease.

## Background

Elective pelvic lymph node irradiation has been suggested to improve progression-free survival in unfavorable intermediate- to high-risk prostate cancer [1–3]. A course of conventionally-fractionated pelvic radiotherapy for prostate cancer is delivered over a period of approximately 9 weeks, which is inconvenient for elderly patients and patients traveling long distances. With implementation of the Radiation Oncology Alternative Payment Model, greater emphasis is placed on cost-effectiveness with more abbreviated treatments to increase access to care. Furthermore, hypofractionated radiotherapy may result in a greater therapeutic ratio for prostate cancer resulting in greater efficacy than conventional fractionation as suggested by the linear quadratic model with a low α/β ratio of 1.5–3.

With improvements in the precision of radiation delivery, several studies have supported the use of moderate hypofractionation (240–340 cGy) directed to the prostate with favorable toxicity profiles [4–6]. In these studies, the clinical target volume consisted of the prostate with or without the seminal vesicles and pelvic lymph nodes were not treated. Limited data is available evaluating the safety and efficacy of hypofractionated pelvic nodal radiotherapy.

The goal of this study was to evaluate the feasibility in terms of clinical outcome and toxicity of a hypofractionated regimen with inclusion of prostate, seminal vesicles, and pelvic lymph node irradiation for the treatment of intermediate- and high-risk prostate cancer in patients with logistical barriers to completing a course of conventionally fractionated pelvic radiotherapy.

## Methods

### Patient selection

Under approval of our institutional review board (IRB), we performed a retrospective chart abstraction to identify men treated with hypofractionated whole pelvic radiotherapy (WPRT). All men had histologically confirmed intermediate- to high-risk prostate adenocarcinoma by NCCN criteria (T1c-T3b N0, PSA > 10, and/or Gleason score ≥ 7) and greater than 15% risk of lymph node involvement by the Roach formula [7]. Patients were offered accelerated, hypofractionated WPRT if there were logistical difficulties with completing five weeks of pelvic radiotherapy, such as lack of transportation or nearby housing. Patients were not offered accelerated, hypofractionated WPRT if they had prior pelvic irradiation, prostate brachytherapy, history of inflammatory bowel disease or major bowel surgery, or prior transurethral resection of the prostate (TURP) procedure. Patients also received androgen deprivation therapy (ADT) at the discretion of the treating radiation oncologist.

### Whole pelvis radiation

The hypofractionated dose of 41.25 Gy in 15 fractions of 2.75 Gy to the elective pelvic nodes, prostate, and seminal vesicles was selected based on the linear-quadratic model to be equivalent to a dose of 45 Gy in 1.8 Gy fractions and using an α/β value of 3 for prostate cancer. The elective pelvic nodal treatment volume included the obturator, external iliac, proximal internal iliac, and common iliac nodes up to the level corresponding to the L4–L5 interspace. The presacral nodes from L5-S1 to S3 were included. The inferior extent of the external iliac nodes was at the top of the femoral heads and the inferior extent of the obturator lymph nodes was at the top of the symphysis pubis. A 5–7 mm PTV margin around the pelvic nodal CTV was used. 2.75 Gy per fraction was used as it was previously used to target the prostate alone to 55 Gy with comparable toxicity with standard fractionation schemes [8]. Intensity modulated radiation therapy with daily image guidance was performed using both cone beam computed tomography with alignment to bony anatomy and prostate fiducial markers.

### Boost treatment to prostate and seminal vesicles

Radiation to the prostate and seminal vesicles was delivered as a sequential boost treatment within 14 days after pelvic radiotherapy portion was completed. The institutional practice was to offer 15 Gy in one fraction with high dose rate brachytherapy. Patients who refused or were not candidates for brachytherapy were offered boost treatment with stereotactic body radiotherapy to a total dose of 19 Gy in 2 fractions of 9.5 Gy. For SBRT, patients were simulated with an empty rectum and empty bladder due to the limited ability of patients to maintain a full bladder through the duration of SBRT treatment. All patients also underwent an MRI of the pelvis/prostate for accurate delineation of the prostate and urethra for treatment planning purposes. SBRT was delivered on a Cyberknife machine with daily imaging guidance using prostate fiducial tracking, including orthogonal pair imaging prior to treatment delivery with matching to prostate fiducials as well as orthogonal pair imaging during treatment delivery to account for intrafraction motion.

### Follow-up

After treatment, patients were followed every 3 months for the initial 2 years, then every 6 months up to year 5, and annually thereafter with prostate specific antigen (PSA) testing. At each follow-up visit, patients completed IPSS and SHIM QOL questionnaires. Toxicity was assessed according to the Common Terminology Criteria for Adverse Events (CTCAE) v5.0.

### Statistical analysis

Descriptive analysis was performed using median and interquartile values for continuous variables. Predictive analysis was performed using logistic regression. Comparator p-values were reported using a two-sample two-sided Mann–Whitney U test. P-values of less than 0.05 were considered to be significant.

## Results

### Patient and tumor characteristics

Among 22 patients, the median age was 71 (Table 1). One (5%), 16 (73%), and 5 (23%) of patients had had clinical T1, T2, and T3 disease, respectively. 19 (86%) of tumors were Gleason grade 7 and 3 (14%) were Gleason grade 9. According to NCCN risk stratification, 13 (59%), 7 (32%), and 2 (9%) of patients had intermediate-, high-, and very high-risk disease, respectively. 21 patients (95%) patients received androgen deprivation therapy (ADT), of whom 19 patients (86%) received a total of 4 months.

**Table 1:** Patient characteristics

Age	
Median (IQR)	71 (68–74)
T-Stage (MRI or TRUS)	
1c	1 (5%)
2a	12 (55%)
2b	1 (5%)
2c	3 (14%)
3a	5 (23%)
Gleason	
3 + 3	1 (5%)
3 + 4	9 (41%)
4 + 3	9 (41%)
4 + 5	1 (5%)
5 + 4	2 (9%)
Pre-treatment PSA	
Median (IQR)	8.8 (5.8–9.5)
NCCN risk group	
Intermediate risk	13 (59%)
High risk	7 (32%)
Very high risk	2 (9%)

### Radiotherapy characteristics

All 22 patients received hypofractionated whole pelvic radiotherapy with intensity modulated radiotherapy (IMRT) to 41.25 Gy in 15 fractions over three weeks (Table 2). 20 (91%) patients received high dose-rate brachytherapy boost with 15 Gy in a single fraction via a single perineal implant procedure. 2 (9%) patients received stereotactic body radiation therapy (SBRT) boost to the prostate with 19 Gy in two fractions on consecutive days.

**Table 2:** Radiotherapy characteristics

Whole Pelvis IMRT	
41.25 Gy in 15 fractions	22 (100%)
Boost	
HDR 15 Gy in one fraction	20 (91%)
SBRT 19 Gy in two fractions	2 (9%)
ADT	
None	1 (5%)
4 months	19 (86%)
6 months	1 (5%)
12 months	1 (5%)

### Whole pelvis radiotherapy parameters

Dose-volume characteristics of hypofractionated whole pelvis radiotherapy is summarized in Tables 3 and 4. The median small bowel V44 Gy (54 Gy at 1.8 Gy/fx) was 0 cc (IQR 0–0.01 cc) and median small bowel V32.4 Gy (40 Gy at 1.8 Gy/fx) was 156 cc (IQR 100–216 cc). The median bladder V37.8 Gy (45 Gy at 1.8 Gy/fx) was 29 cc (IQR 20–49 cc) and 17% of the total bladder volume (IQR 13–21%). The median rectum V37.8 Gy was 5 cc (IQR 3–9 cc) and 7% of the total rectal volume (IQR 4–10%). There was no correlation found between dosimetric parameters and grade ≥ 2 GU or GI toxicity.

**Table 3:** Whole pelvis radiotherapy dosimetric parameters

Organ	Parameter (Gy)	Parameter (equivalent at 1.8 Gy per fraction)	Median volume (IQR)	Median % of total organ volume (IQR)
Bowel	V44	D54 Gy	0 cc (0–0.01 cc)	
Bowel	V32.4	D40 Gy	156 cc (100–216 cc)	
Bladder	V37.8	D45 Gy	29 cc (20–49 cc)	17% (13–21%)
Rectum	V37.8	D45 Gy	5 cc (3–9 cc)	7% (4–10%)

**Table 4:** Dosimetry

Patient	Bowel	Bowel	Bladder	Bladder	Rectum	Rectum	Penile Bulb Mean Dose (Gy)
	V44Gy (cc)	V32.4 Gy (cc)	V37.8 Gy (% of total volume)	V37.8 Gy (cc)	V37.8 Gy (%) of total volume	V37.8 Gy (cc)	
1	0.67	154.82	18.94	38.56	5.52	2.6	20.35
2	0	81.14	9.81	23.43	5.57	3.33	21.2
3	0	102.89	13.12	14.84	2.32	1.84	15.8
4	0	110.89	18.58	50.07	4.56	2.81	40.94
5	0	188.28	12.41	20.41	12.08	11.07	28.86
6	0.01	98.58	12.34	15.81	2.98	1.63	9.547
7	0	253.34	20.03	34.62	13.69	15.22	38.35
8	0.24	216.46	25.29	89.23	2.19	1.64	9.76
9	0.01	318.92	23.06	57.01	6.59	5.93	20.66
10	0	162.8	13.63	47.1	3.92	2.92	15.2
11	0	114.74	19.72	21.03	16.13	12.25	22.61
12	0	220.75	27.69	84.98	10.18	5.16	19.42
13	0	250.2	20.67	23.27	7.04	4.92	6.53
14	0	137.09	6.38	10.98	4.25	2.77	13.99
15	0.03	157.55	17.15	42.92	8.12	5.74	22.51
16	0	20.51	26.24	17.92	21.57	14.71	3.81
17	0	25.59	16.6	67.46	11.22	9.33	11.06
18	0	44.09	9.5	16.86	8.18	14.25	18.00
19	0	34.18	9.73	38.04	7.28	6.29	4.51
20	0	214.57	14.92	20.1	0	0	3.30
21	0.01	164.44	13.95	23.02	5.82	6.34	11.30
22	7.64	289.5	25.42	54.37	9.18	9.31	9.09

### Disease control and toxicity

**Fig. 1:**
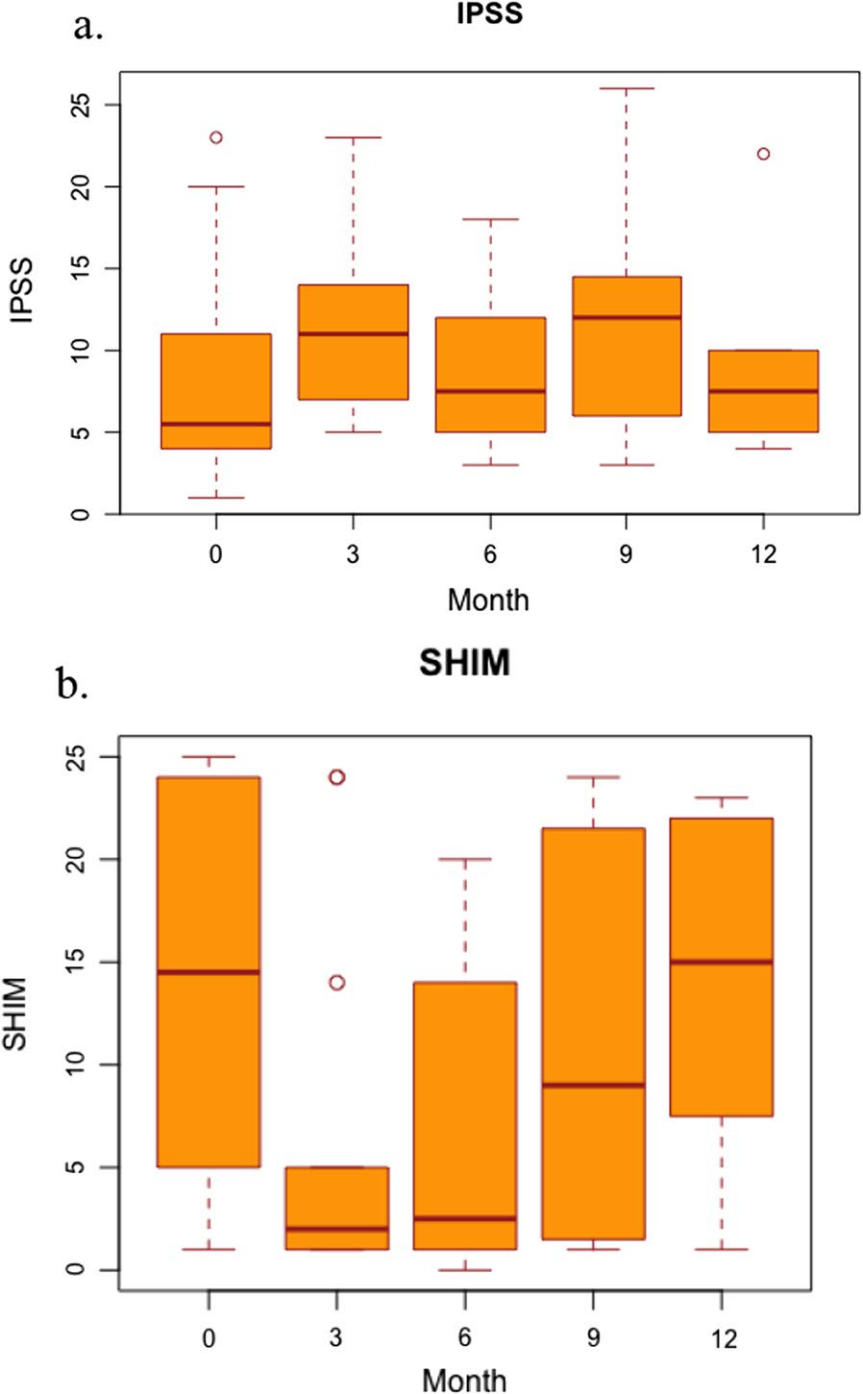
Patient reported quality of life. IPSS = international prostate symptom score (IPSS); SHIM = showing sexual health inventory in men (SHIM)

At a median follow-up of 32 months, there were no biochemical failures, and the median post-treatment PSA was 0.09 (range 0–1.28). The crude rates of acute grade 2 or higher GI and GU toxicities were 23% and 23% (Table 5). These toxicities mostly consisted of urinary frequency, urgency, and diarrhea. The late or persistent grade 2 or higher GI and GU toxicity rates were 4% and 13%, consisting of diarrhea and urinary frequency. No urinary strictures were observed. There was a trend of worsening patient-reported IPSS (*p* = 0.08) and SHIM scores (*p* = 0.06) at 3-month follow-up but no significant difference by 12-month follow-up (Fig. 1). An initial decline in SHIM scores may be attributed, in part, by the use of androgen deprivation therapy in our patient cohort.

**Table 5:** Toxicities

Patient	GI (Grade)		GU (Grade)	
	Acute	Late	Acute	Late
1	0	0	0	0
2	1	0	0	0
3	2	2	1	1
4	0	0	0	0
5	2	0	2	2
6	1	0	1	0
7	1	0	2	0
8	0	0	2	2
9	0	0	0	0
10	0	0	1	1
11	0	0	1	1
12	0	0	0	0
13	1	0	2	0
14	0	0	0	0
15	2	0	2	0
16	0	0	0	0
17	0	0	0	0
18	1	0	0	0
19	2	0	1	2
20	0	0	1	0
21	2	0	0	0
22	0	0	1	0
Number of patients with Grade ≥ 2	5	1	5	3
% patients with Grade ≥ 2	23	5	23	14

## Discussion

Data is limited on the use of hypofractionated radiotherapy for elective treatment of pelvic nodes in patients at increased risk of harboring occult nodal disease. Our study demonstrates a 3 year biochemical free survival of 100% with a median post-treatment PSA was 0.09 and no acute or late grade ≥ 3 GU or GI toxicity. No difference in patient-reported urinary or sexual quality of life was noted pre- and 12 months post-treatment.

Hypofractionation may improve the therapeutic ratio of EBRT in localized prostate cancer as the alpha–beta ratios of nearby organs-at-risk are considered to be higher than prostate cancer [9]. Moderately hypofractionated radiotherapy without lymph node irradiation is well-studied, both in terms of efficacy and similar toxicity profiles, compared to conventional fractionation for the treatment of localized prostate cancer [3, 4, 10–15]. Nevertheless, there remains caution in the applicability of these regimens for all patients with localized prostate cancer, in part due to the relatively short follow-up of some studies and given the concern that certain subgroups have been observed to have more toxicity with hypofractionation. Datta et al. performed a meta-analysis of ten phase III trials evaluating conventional versus hypofractionated radiotherapy and found that acute GI toxicity was worse with the use of ADT or full inclusion of the seminal vesicles [16]. Brenner et al. commented that moderate hypofractionation regimens of higher BED were associated with worse toxicity [17].

The toxicity profile of hypofractionated radiotherapy with lymph node irradiation in higher risk prostate cancer is much less studied to date [18]. Norkus et al. reported a randomized controlled trial that tested a WPRT fractionation of 44 Gy in 2.2 Gy per fraction [19]. WPRT of 25 Gy in 5 Gy per fraction was studied in both the SATURN and FASTR trials with discordant outcomes as the former reported no grade 3 or higher toxicities while the latter reported 26% grade 3 or higher GI or GU toxicities at 6 months [20, 21]. The experimental arm in the ongoing PRIME Trial (NCT03561961) is also studying a WPRT regimen of 25 Gy in 5 Gy per fraction. To our knowledge, there are no reports on the safety of moderately-fractionated whole pelvis radiotherapy in localized prostate cancer with this fractionation scheme.

The 22 patients in our study were observed to have acute grade 2 GI and GU toxicities of 23% and 23%, respectively. The late grade 2 GI and GU toxicities were 4% and 13%, respectively. No grade 3 or higher acute or late toxicities were observed in the current study. The low toxicity is favorable when compared to RTOG 0321, which utilized HDR brachytherapy boost conventionally fractionated external beam radiation, and prior studies of HDR brachytherapy boost with hypofractionated EBRT to the prostate and seminal vesicles without pelvic radiation [22]. Shahid et al. and Tharmalingam et al. have both reported very low rates of grade 3 or higher GI and GU toxicities in of 125 and 411 patients treated with a 15 Gy single fraction HDR boost with 37.5 Gy in 2.5 Gy per fraction of EBRT to the prostate and seminal vesicles after roughly 5 years of follow-up [23, 24]. Furthermore, a recent phase IB study combining a single 15 Gy HDR brachytherapy boost with 5, 7, or 10 fractions of hypofractionated EBRT to the prostate and proximal seminal vesicles reported only one acute grade 3 GI and GU toxicity but no late grade 3 GU or GI toxicities at a median follow-up of 36 months [25]. Similarly, a phase II study evaluating LDR brachytherapy in combination with 25 Gy in 5 fractions of external beam radiotherapy to the prostate and seminal vesicles similarly demonstrated low rates of toxicity [26]. Altogether, the low GU and GI toxicity observed in the current study with hypofractionated pelvic nodal irradiation is in line with the toxicity rates observed from published studies evaluating hypofractionated treatment to the prostate and seminal vesicles only.

The implementation of the Radiation Oncology Alternative Payment Model has placed greater emphasis on cost-effectiveness with more abbreviated treatments. When compared to long courses of conventionally fractionated EBRT, both HDR brachytherapy and hypofractionated EBRT offer two cost effective treatments that have shown promising effectiveness with low toxicity rates. This could potentially allow for greater accessibility to oncologic care and providesignificant value while striving for cost-effective healthcare.

The strengths of our study include close and detailed follow up for each patient with toxicities reported on an individual basis. Patient reported outcomes, including IPSS and SHIM scores, were documented at 3 month intervals after completion of therapy, which demonstrated fluctuations in patient reported quality of life. However, limitations to this study include its retrospective nature, limited follow up time, and small cohort size. Thus, long term control and toxicity data is not available with our cohort.

## Conclusion

Moderate hypofractionated whole pelvis radiotherapy in combination with HDR brachytherapy or SBRT boost is a promising treatment option for patients at risk of harboring occult nodal disease. Thus, this may serve as a basis for a larger randomized controlled study.

## Data Availability

The datasets used/analyzed during the current study are available from the corresponding author on reasonable request.

## Abbreviations

ADT: Androgen deprivation therapy
CTCAE: Common terminology criteria for adverse events
HDR: High dose rate
IMRT: Intensity modulated radiotherapy
PSA: Prostate specific antigen
SBRT: Stereotactic body radiation therapy
TURP: Transurethral resection of the prostate
WPRT: Whole pelvic radiation therapy

## References

[1] Roach M, DeSilvio M, Valicenti R, Grignon D, Asbell SO, Lawton C (2006). Whole-pelvis, “mini-pelvis”, or prostate-only external beam radiotherapy after neoadjuvant and concurrent hormonal therapy in patients treated in the radiation therapy oncology group 9413 trial. Int J Radiat Oncol Biol Phys.

[2] Mantini G, Tagliaferri L, Mattiucci GC, Balducci M, Frascino V, Dinapoli N (2011). Effect of whole pelvic radiotherapy for patients with locally advanced prostate cancer treated with radiotherapy and long-term androgen deprivation therapy. Int J Radiat Oncol Biol Phys.

[3] Murthy V, Maitre P, Kannan S, Panigrahi G, Krishnatry R, Bakshi G (2021). Prostate-only versus whole-pelvic radiation therapy in high-risk and very high-risk prostate cancer (POP-RT): outcomes from phase III randomized controlled trial. J Clin Oncol.

[4] Catton CN, Lukka H, Gu CS, Martin JM, Supiot S, Chung PWM (2017). Randomized trial of a hypofractionated radiation regimen for the treatment of localized prostate cancer. J Clin Oncol.

[5] Dearnaley D, Syndikus I, Mossop H, Khoo V, Birtle A, Bloomfield D (2016). Conventional versus hypofractionated high-dose intensity-modulated radiotherapy for prostate cancer: 5-year outcomes of the randomised, non-inferiority, phase 3 CHHiP trial. Lancet Oncol.

[6] Incrocci L, Wortel RC, Alemayehu WG, Aluwini S, Schimmel E, Krol S (2016). Hypofractionated versus conventionally fractionated radiotherapy for patients with localised prostate cancer (HYPRO): final efficacy results from a randomised, multicentre, open-label, phase 3 trial. Lancet Oncol.

[7] Roach M, Marquez C, Yuo HS, Narayan P, Coleman L, Nseyo UO (1994). Predicting the risk of lymph node involvement using the pre-treatment prostate specific antigen and gleason score in men with clinically localized prostate cancer. Int J Radiat Oncol Biol Phys.

[8] Yeoh EE, Botten RJ, Butters J, di Matteo AC, Holloway RH, Fowler J (2011). Hypofractionated versus conventionally fractionated radiotherapy for prostate carcinoma: Final results of phase III randomized trial. Int J Radiat Oncol Biol Phys.

[9] Morgan SC, Hoffman K, Loblaw DA, Buyyounouski MK, Patton C, Barocas D (2018). Hypofractionated radiation therapy for localized prostate cancer: executive summary of an ASTRO, ASCO, and AUA evidence-based guideline. Pract Radiat Oncol.

[10] Dearnaley D, Hall E (2016). Hypofractionated radiotherapy for prostate cancer—Authors’ reply. Lancet Oncol.

[11] Arcangeli G, Saracino B, Arcangeli S, Gomellini S, Petrongari MG, Sanguineti G (2017). Moderate hypofractionation in high-risk, organ-confined prostate cancer: final results of a phase III randomized trial. J Clin Oncol.

[12] de Vries KC, Wortel RC, Oomen-de Hoop E, Heemsbergen WD, Pos FJ, Incrocci L (2020). Hyprofractionated versus conventionally fractionated radiation therapy for patients with intermediate- or high-risk, localized, prostate cancer: 7-year outcomes from the randomized, multicenter, open-label, phase 3 HYPRO trial. Int J Radiat Oncol Biol Phys.

[13] Avkshtol V, Ruth KJ, Ross EA, Hallman MA, Greenberg RE, Price RA (2020). Ten-year update of a randomized, prospective trial of conventional fractionated versus moderate hypofractionated radiation therapy for localized prostate cancer. J Clin Oncol.

[14] Lee WR, Dignam JJ, Amin MB, Bruner DW, Low D, Swanson GP (2016). Randomized phase III noninferiority study comparing two radiotherapy fractionation schedules in patients with low-risk prostate cancer. J Clin Oncol.

[15] Hoffman KE, Voong KR, Levy LB, Allen PK, Choi S, Schlembach PJ (2018). Randomized trial of hypofractionated, dose-escalated, intensity-modulated radiation therapy (IMRT) versus conventionally fractionated IMRT for localized prostate cancer. J Clin Oncol.

[16] Datta NR, Stutz E, Rogers S, Bodis S (2017). Conventional versus hypofractionated radiation therapy for localized or locally advanced prostate cancer: a systematic review and meta-analysis along with therapeutic implications. Int J Radiat Oncol Biol Phys.

[17] Brenner DJ, Hall EJ (2018). Are we now able to define guidelines for moderate hypofractionation in prostate cancer radiation therapy?. Int J Radiat Oncol Biol Phys.

[18] Kaidar-Person O, Roach M, Créhange G (2013). Whole-pelvic nodal radiation therapy in the context of hypofractionation for high-risk prostate cancer patients: a step forward. Int J Radiat Oncol Biol Phys.

[19] Norkus D, Karklelyte A, Engels B, Versmessen H, Griskevicius R, de Ridder M (2013). A randomized hypofractionation dose escalation trial for high risk prostate cancer patients: interim analysis of acute toxicity and quality of life in 124 patients. Radiat Oncol.

[20] Musunuru HB, D’Alimonte L, Davidson M, Ho L, Cheung P, Vesprini D (2018). Phase 1–2 study of stereotactic ablative radiotherapy including regional lymph node irradiation in patients with high-risk prostate cancer (SATURN): early toxicity and quality of life. Int J Radiat Oncol Biol Phys.

[21] Bauman G, Ferguson M, Lock M, Chen J, Ahmad B, Venkatesan VM (2015). A phase 1/2 trial of brief androgen suppression and stereotactic radiation therapy (FASTR) for high-risk prostate cancer. Int J Radiat Oncol Biol Phys.

[22] Hsu IC, Rodgers JP, Shinohara K, Purdy J, Michalski J, Roach M (2020). Long-term results of NRG oncology/RTOG 0321: a phase II trial of combined high dose rate brachytherapy and external beam radiation therapy for adenocarcinoma of the prostate. Int J Radiat Oncol Biol Phys.

[23] Shahid N, Loblaw A, Chung HT, Cheung P, Szumacher E, Danjoux C (2017). Long-term toxicity and health-related quality of life after single-fraction high dose rate brachytherapy boost and hypofractionated external beam radiotherapy for intermediate-risk prostate cancer. Clin Oncol.

[24] Tharmalingam H, Tsang Y, Choudhury A, Alonzi R, Wylie J, Ahmed I (2020). External beam radiation therapy (EBRT) and high-dose-rate (HDR) brachytherapy for intermediate and high-risk prostate cancer: the impact of EBRT volume. Int J Radiat Oncol Biol Phys.

[25] Den RB, Greenspan J, Doyle LA, Harrison AS, Peng C, Williams NL (2020). A phase IB clinical trial of 15 Gy HDR brachytherapy followed by hypofractionated/SBRT in the management of intermediate-risk prostate cancer. Brachytherapy.

[26] Kollmeier MA, McBride S, Varghese M, Debonis D, Zhang Z, Cohen G (2020). Low-dose-rate brachytherapy combined with ultrahypofractionated radiation therapy for clinically localized, intermediate-risk prostate cancer: results from a prospective trial. Int J Radiat Oncol Biol Phys.

